
JOURNAL INFORMATION
==============================
NLM Title Abbreviation: J Diabetes Investig
Journal ID: J Diabetes Investig
NLM Journal ID: 101520702
Journal ID: JDI

Journal of Diabetes Investigation

ISSN: 2040-1116
EISSN: 2040-1124
Publisher: Wiley

ARTICLE INFORMATION
==============================
PMCID: PMC11474639
DOI: 10.1111/jdi.14155
Article ID: JDI14155
Article version: 1
Subjects: Issue Information, Issue Information

Issue Information

Electronic publication date: 2024 Jan 23
Publication date: 2024 Feb
Volume: 15
Issue: 2
First page: 139
Last page: 140
Copyright: © 2024 The Authors. Journal of Diabetes Investigation published by Asian Association for the Study of Diabetes (AASD) and John Wiley & Sons Australia, Ltd
License: This is an open access article under the terms of the http://creativecommons.org/licenses/by/4.0/ License, which permits use, distribution and reproduction in any medium, provided the original work is properly cited.
License URL: https://creativecommons.org/licenses/by/4.0/

Figure count: 0
Table count: 0
Page count: 5

source-schema-version-number: 2.0
cover-date: February 2024
details-of-publishers-convertor: Converter:WILEY_ML3GV2_TO_JATSPMC version:6.4.9 mode:remove_FC converted:15.10.2024

==============================

JDI

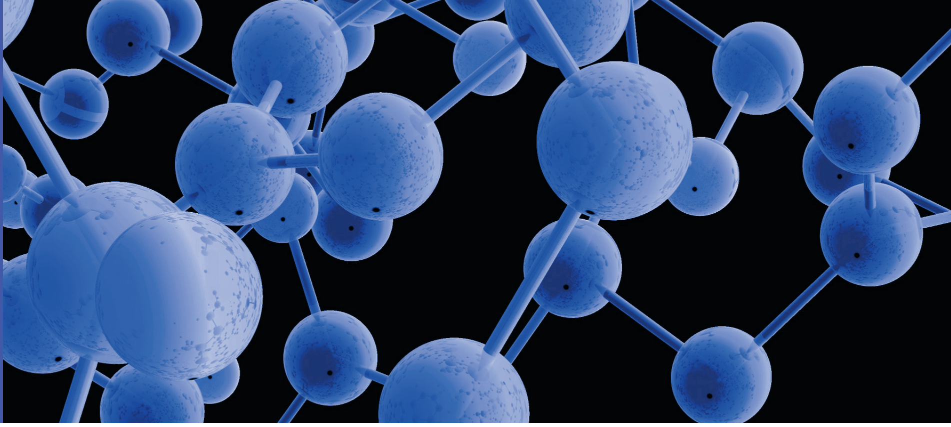

Volume 15 | Issue 2
February 2024

### Journal of Diabetes Investigation

Official Journal of the Asian Association for the Study of Diabetes

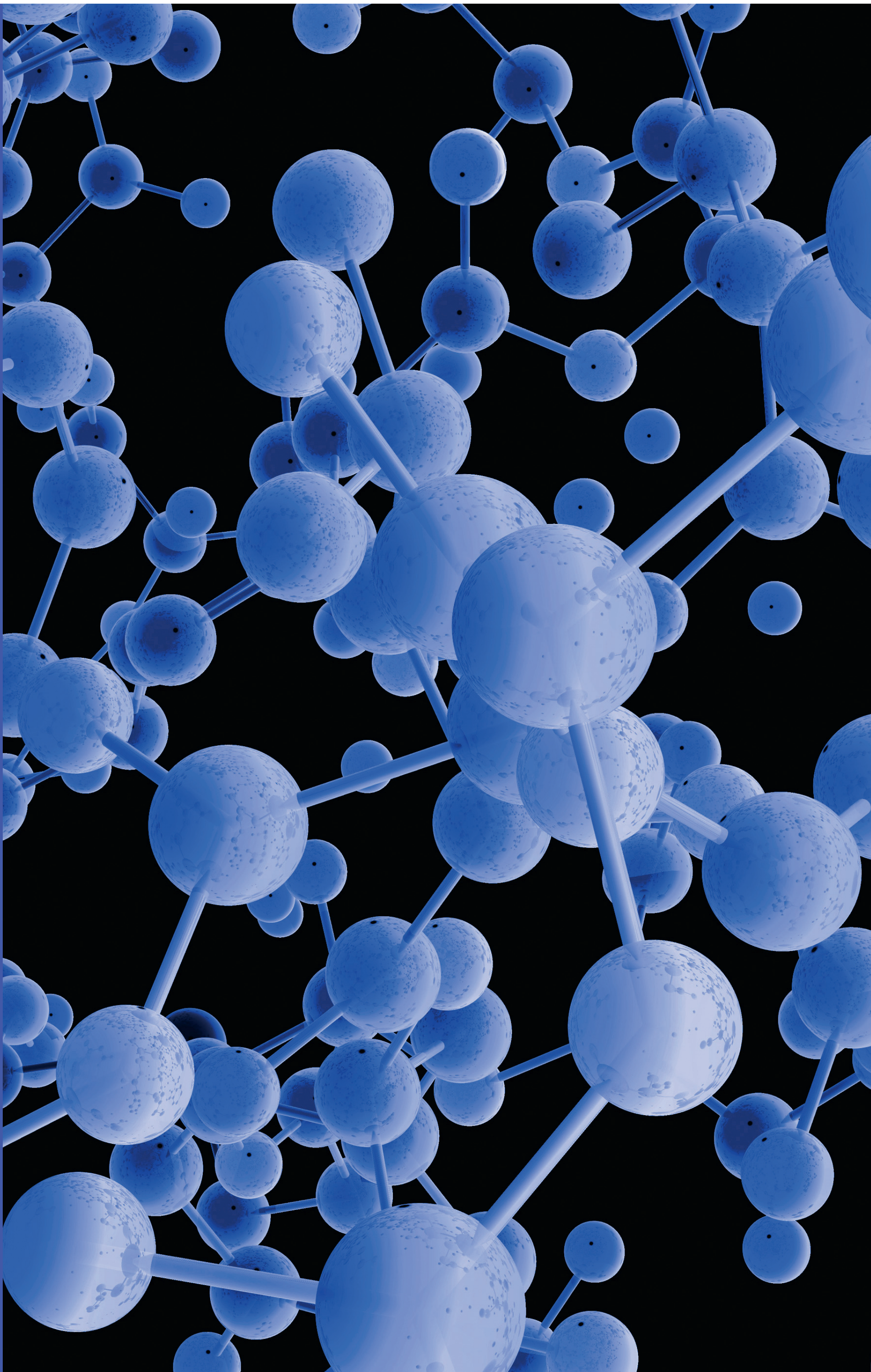

Prevention of onset of diabetes

THE ASIAN ASSOCIATION FOR THE STUDY OF DIABETES
AASD
WILEY

JDI Journal of Diabetes Investigation
Open access
Official Journal of the Asian Association for the Study of Diabetes

**Editor-in-Chief**
Nigishi Hotta, *Japan Organization of Occupational Health and Safety, Chubu Rosai Hospital, Nagoya, Japan*

**Co-Editor**
Domenico Accili, *Columbia University, New York, USA*

**Expert Editor**
Bo Ahren, *Lund, Sweden*
Young Min Cho, *Seoul, Korea*
Sean Dinneen, *Galway, Ireland*
Marc Gilbert, *Montpelier, France*
Limei Liu, *Shanghai, China*
Ronald Ma, *Hong Kong, China*
Louis Philipson, *Chicago, USA*
Wayne H-H Sheu, *Taichung, Taiwan*
Bernard Thorens, *Lausanne, Switzerland*
Daisuke Yabe, *Gifu, Japan*

**Editorial Board**
Eiichi Araki, *Kumamoto, Japan*
Xiaoping Chen, *Beijing, China*
Lee-Ming Chuang, *Taipei, Taiwan*
Masakazu Haneda, *Asahikawa, Japan*
Larry Low-Tone Ho, *Taipei, Taiwan*
Tianpei Hong, *Beijing, China*
Akihisa Imagawa, *Osaka, Japan*
Nobuya Inagaki, *Kyoto, Japan*
Linong Ji, *Beijing, China*
Weiping Jia, *Shanghai, China*
Yi-Der Jiang, *Taipei, Taiwan*
Takashi Kadowaki, *Tokyo, Japan*
Kohei Kaku, *Kurashiki, Japan*
Keizo Kanasaki, *Izumo, Japan*
Doo-Man Kim, *Seoul, Korea*
Karen Lam, *Hong Kong, China*
Hong Kyu Lee, *Gyeonggi-do, Korea*
In-Kyu Lee, *Daegu, Korea*
Moon-Kyu Lee, *Seoul, Korea*
Jiro Nakamura, *Nagakute, Japan*
Kishio Nanjo, *Wakayama, Japan*
Wataru Ogawa, *Kobe, Japan*
Yutaka Seino, *Osaka, Japan*
Shyi-Jang Shin, *Kaohsiung, Taiwan*
Hirohito Sone, *Niigata, Japan*
Kathryn Tan, *Hong Kong, China*
Yukio Tanizawa, *Ube, Japan*
Kohjiro Ueki, *Tokyo, Japan*
Jun Wada, *Okayama, Japan*
Hirotaka Watada, *Tokyo, Japan*
Jianping Weng, *Guangzhou, China*
Soroku Yagihashi, *Hirosaki, Japan*
Hiroshi Yamamoto, *Kanazawa, Japan*
Toshimasa Yamauchi, *Tokyo, Japan*
Wenying Yang, *Beijing, China*
Kazuki Yasuda, *Tokyo, Japan*
Koutaro Yokote, *Chiba, Japan*
Kun-Ho Yoon, *Seoul, Korea*
Dalong Zhu, *Nanjing, China*

**International Editorial Board**
Michael A. Brownlee, *New York, USA*
Subrata Chakrabarti, *Ontario, Canada*
Mark E. Cooper, *Prahran, Australia*
Philippe Froguel, *London, UK*
Wilfred Fujimoto, *Hawaii, USA*
Michael S. German, *San Francisco, USA*
Andrew Hattersley, *Exeter, UK*
Jean-Claude Henquin, *Brussels, Belgium*
Jens Juul Holst, *Copenhagen, Denmark*
Frank B. Hu, *Boston, USA*
C. Ronald Kahn, *Boston, USA*
George L. King, *Boston, USA*
Rayaz A. Malik, *Manchester, UK*
Michael Mauer, *Minneapolis, USA*
Carl Erik Mogensen, *Aarhus, Denmark*
Steven E. Shoelson, *Boston, USA*
Jay S. Skyler, *Miami, USA*
Paul Zimmet, *Melbourne, Australia*

**Assistant Editorial Board**
Tien-Jyun Chang, *Taipei, Taiwan*
Yoshiyuki Hamamoto, *Osaka, Japan*
Norio Harada, *Kyoto, Japan*
Yi-Jen Hung, *Taipei, Taiwan*
Chi-Min Hwu, *Taipei, Taiwan*
Hideki Kamiya, *Aichi, Japan*
Dae Jung Kim, *Suwon, Korea*
Seung-Hwan Lee, *Seoul, Korea*
Hung-Yuan Li, *Taipei, Taiwan*
Takashi Matsuzaka, *Tsukuba, Japan*
Keiko Naruse, *Aichi, Japan*
Takeshi Nishikawa, *Kumamoto, Japan*
Eung-Jung Rhee, *Seoul, Korea*
Yusuke Seino, *Aichi, Japan*
Hironori Waki, *Tokyo, Japan*

**Aims and Scope:** *Journal of Diabetes Investigation* is your core diabetes journal from Asia; the official journal of the Asian Association for the Study of Diabetes (AASD). The journal publishes original research, country reports, commentaries, reviews, mini-reviews, case reports, letters, as well as editorials and news. The journal embraces clinical and experimental research in diabetes and related areas. This includes aspects of prevention, treatment, as well as molecular aspects and pathophysiology. Translational research focused on the exchange of ideas between clinicians and researchers is also welcome.

**Address for Editorial Correspondence:**
The Editor, *Journal of Diabetes Investigation*, Nomura Fudosan Nishi Shinjuku Building, 8F 8-4-2 Nishi Shinjuku, Shinjuku-ku, Tokyo 160-0023, Japan. Tel: +81 3 4520 9009; Fax: +81 3 4520-9059 Email: JDI@wiley.com

**Copyright © 2024 Asian Association for the Study of Diabetes and John Wiley & Sons Australia, Ltd**

**Cover:** Insulin molecule © Pasieka/Science Photo Library

This journal is available online at Wiley Online Library. Visit wileyonlinelibrary.com to search the articles and register for table of contents and e-mail alerts.

**For submission instructions, and all other information visit wileyonlinelibrary.com/journal/jdi**

ISSN 2040-1124 (Online)

JDI.JEB.Feb24

WILEY

**Publisher**
*Journal of Diabetes Investigation* is published by John Wiley & Sons Australia, Ltd
Level 19, 15 William Street,
Melbourne, Victoria 3000,
Australia.
Tel: +61 (0)3 9274 3100

**Production Editor**
Jayalakshmi Mohan (email: JDIproduction@wiley.com)

**Abstracting and Indexing Services**
The Journal is indexed by Academic OneFile (GALE Cengage), Biological Abstracts (Clarivate Analytics), Biological Science Database (ProQuest), BIOSIS Previews (Clarivate Analytics), Embase (Elsevier), Health & Medical Collection (ProQuest), Health Research Premium Collection (ProQuest), Hospital Premium Collection (ProQuest), MEDLINE/PubMed (NLM), Natural Science Collection (ProQuest), ProQuest Central (ProQuest), Science Citation Index Expanded (Clarivate Analytics), SciTech Premium Collection (ProQuest), SCOPUS (Elsevier), Web of Science (Clarivate Analytics).

**Open Access and Copyright**
All articles published by *Journal of Diabetes Investigation* are fully open access: immediately freely available to read, download and share. All *Journal of Diabetes Investigation* articles accepted from 24 September 2013 are published under the terms of the Creative Commons Attribution 4.0 Unported (CC BY 4.0) license, which permits use, distribution and reproduction in any medium, provided the original work is properly cited. Articles accepted before this date were published under the agreement as stated in the final article. Copyright on any *Journal of Diabetes Investigation* article accepted after 24 September 2013, the date on which *Journal of Diabetes Investigation* became an open access journal, is retained by the author(s). Authors grant to *Journal of Diabetes Investigation* and Wiley a license to publish the article and for Wiley to identify itself as the original publisher. Authors also grant any third party the right to use the article freely as long as its integrity is maintained and its original authors, citation details and publisher are identified. Please visit https://authorservices.wiley.com/author-resources/Journal-Authors/open-access/hybrid-open-access.html for further information about Open Access.

**Use by commercial ''for-profit'' organisations (not applicable to CC BY 4.0 licensed articles)**
Use of Wiley Open Access articles for commercial, promotional, or marketing purposes requires further explicit permission from Wiley and will be subject to a fee. Requests to reproduce material from this journal content are being handled through the Rightslink® online service.

- Locate the article you wish to reproduce on Wiley Online Library (http://onlinelibrary.wiley.com)
- Navigate to the abstract page
- Click on the 'Request Permissions' link, under the 'ARTICLE TOOLS' menu
- Follow the online instructions and select your requirements from the drop down options and click on 'quick price' to get a quote
- Create a RightsLink® account to complete your transaction (and pay, where applicable)
- Read and accept our Terms & Conditions and Download your license
- For any technical queries please contact customercare@copyright.com
- For further information and to view a Rightslink® demo please visit www.wiley.com and select Rights & Permissions.
- Print reprints of Wiley Open Access articles can be purchased from corporatesales@wiley.com.

**Disclaimer**
The Publisher, the Asian Association for the Study of Diabetes, and Editors cannot be held responsible for any errors in or any consequences arising from the use of information contained in this journal. The views and opinions expressed do not necessarily reflect those of the Publisher, the Asian Association for the Study of Diabetes, Editors, or Authors neither does the publication of advertisements constitute any endorsement by the Publisher, the Asian Association for the Study of Diabetes, or Editors of the products advertised.

Wiley Open Access articles posted to repositories or websites are without warranty from Wiley of any kind, either express or implied, including, but not limited to, warranties of merchantability, fitness for a particular purpose, or noninfringement.
To the fullest extent permitted by law Wiley disclaims all liability for any loss or damage arising out of, or in connection, with the use of or inability to use the content.

**Journal Customer Services:** For ordering information, claims and any enquiry concerning your journal subscription please visit our Online Customer Help at https://wolsupport.wiley.com/s/contactsupport or contact your nearest office.
**Americas:** Email: cs-journals@wiley.com; Tel: +1 877 762 2974
**Europe, Middle East and Africa:** Email: cs-journals@wiley.com; Tel: +44 (0) 1243 684083.
**Germany, Austria, Switzerland, Luxembourg, Liechtenstein:** cs-germany@wiley.com; Tel: 0800 1800 536 (Germany)
**Asia Pacific:** Email: cs-journals@wiley.com; Tel: +65 3165 0890.
**Japan:** For Japanese speaking support, Email: cs-japan@wiley.com; .
**For a full list of frequently asked questions,** please visit https://wolsupport.wiley.com/s/

Wiley's Corporate Citizenship initiative seeks to address the environmental, social, economic, and ethical challenges faced in our business and which are important to our diverse stakeholder groups. Since launching the initiative, we have focused on sharing our content with those in need, enhancing community philanthropy, reducing our carbon impact, creating global guidelines and best practices for paper use, establishing a vendor code of ethics, and engaging our colleagues and other stakeholders in our efforts. Follow our progress at www.wiley.com/go/citizenship

VOLUME 15 – ISSUE 2 – FEBRUARY 2024

### Journal of Diabetes Investigation

Open access

### Commentary

**Dipeptidyl peptidase-4 inhibition targeting vascular senescence as a novel treatment for atherosclerosis**
I-Weng Yen and Hung-Yuan Li **143**

### Original Articles

### Basic Science and Research

**MiR-488-3p facilitates wound healing through CYP1B1-mediated Wnt/β-catenin signaling pathway by targeting MeCP2**
Chenchen Zuo, Pengju Fan, Ying Yang, and Chengjun Hu **145**

**Circ_0068087 knockdown attenuates high-glucose-induced human tubular epithelial cell injury in a microribonucleic acid/progestin and adipoQ receptor 3-dependent manner in diabetic nephropathy**
Shu-yan Liu, Hong Wang, Bo Yang, Baohua Hou, Li-sha Sun, Hui Pang, Hui-hui Wang, and Yan-ping Fan **159**

### Short Report

### Clinical Science and Care

**Delayed dinnertime impairs glucose tolerance in healthy young adults**
Fuzuki Nakamura, Yuki Shimba, Saki Toyonaga, Chiemi Kuroda, Asuka Yamato, Yuhi Yamada, and Toshio Hosaka **172**

### Original Articles

**Characteristics of individuals who developed type 2 diabetes from prediabetes despite undergoing interventions, and evaluation of the performance of urinary myo-inositol as a risk factor for developing diabetes**
Fumio Yamagata, Rei Ogata, Yoko Koda, Mayumi Yamamoto, Atsuko Suga, Noriko Kanauchi, Takuji Kohzuma, and Shoji Kawazu **177**

**Associations of age-adjusted coefficient of variation of R-R intervals with autonomic and peripheral nerve function in non-elderly persons with diabetes**
Kazuhiro Sugimoto, Hirozumi Miyaoka, Takashi Sozu, Naohiro Sekikawa, Ryota Wada, Yuko Watanabe, Akira Tamura, Toshiro Yamazaki, Setsu Ohta, and Susumu Suzuki **186**

**Relationship between elevated circulating thrombospondin-1 levels and vascular complications in diabetes mellitus**
Na Guo, Linlin Yang, Xiaozheng Wan, Dongze Qiu, Wenwen Sun, and Huijuan Ma **197**

**The association between metabolic syndrome and insulin resistance with risk of cardiovascular events in different states of cardiovascular health status**
Somayeh Hosseinpour-Niazi, Siamak Afaghi, Parto Hadaegh, Maryam Mahdavi, Hossein Farhadnejad, Maryam Tohidi, Parvin Mirmiran, Fereidoun Azizi, and Farzad Hadaegh **208**

### Clinical Trial

**Effects of hybrid closed-loop system on glycemic control and psychological aspects in persons with type 1 diabetes treated with sensor-augmented pump: A prospective single-center observational study**
Tomoaki Akiyama, Tadashi Yamakawa, Kazuki Orime, Masahiro Ichikawa, Marina Harada, Takumi Netsu, Ryoichi Akamatsu, Keita Nakamura, Satoru Shinoda, and Yasuo Terauchi **219**

### Original Articles

**Mandatory monthly outpatient visits could improve therapeutic inertia in patients with poorly controlled type 2 diabetes**
Ying-Ying Tsai, Ting-Yu Kuo, Meng-Hung Lin, Feng-Chih Shen, and Yung-Hsiang Lin **227**

**The association between alcohol drinking and glycemic management among people with type 2 diabetes in China**
Qing Ye, Xiaojun Ouyang, Zhenzhen Qin, Yijia Chen, Xuening Zhang, Ye Liu, Qinglin Lou, and Fei Xu **237**

### Letter to the Editor

**Longitudinal association of sedentary time with diabetes mellitus and markers of glucose metabolism in middle-aged and older adults: The Hisayama Study**
Susumu Tsuda, Takanori Honda, Mayu Higashioka, Jun Hata, Toshiaki Nakano, Takanari Kitazono, and Toshiharu Ninomiya **245**

### Special Research Reports

**Prevalence and characteristics of diabetic symmetric sensorimotor polyneuropathy in Japanese patients with type 2 diabetes: The Japan Diabetes Complication and its Prevention Prospective study (JDCP study 10)**
Hideki Kamiya, Tatsuhito Himeno, Atsuko Watarai, Masayuki Baba, Rimei Nishimura, Naoko Tajima, and Jiro Nakamura **247**

**New diagnostic criteria (2023) for slowly progressive type 1 diabetes (SPIDDM): Report from Committee on Type 1 Diabetes of the Japan Diabetes Society (English version)**
Akira Shimada, Eiji Kawasaki, Norio Abiru, Takuya Awata, Yoichi Oikawa, Haruhiko Osawa, Hiroshi Kajio, Junji Kozawa, Kazuma Takahashi, Daisuke Chujo, Shinsuke Noso, Tomoyasu Fukui, Junnosuke Miura, Kazuki Yasuda, Hisafumi Yasuda, Akihisa Imagawa, and Hiroshi Ikegami **254**
